# How are hygiene programmes designed in crises? Qualitative interviews with humanitarians in the Democratic Republic of the Congo and Iraq

**DOI:** 10.1186/s13031-022-00476-8

**Published:** 2022-09-02

**Authors:** Sian White, Thomas Heath, Anna C. Mutula, Robert Dreibelbis, Jennifer Palmer

**Affiliations:** 1 Department of Disease Control, London School of Hygiene and Tropical Medicine, London, UK; 2 Independent Consultant, Goma, Democratic Republic of the Congo; 3 Action Contre La Faim, Paris, France; 4 Department of Global Health and Development, London School of Hygiene and Tropical Medicine, London, UK

**Keywords:** Programme design, Humanitarian systems, Localisation of aid, Evidence-based practice, Hygiene

## Abstract

**Background:**

Hygiene behaviour change programmes are complex to design. These challenges are heightened during crises when humanitarian responders are under pressure to implement programmes rapidly despite having limited information about the local situation, behaviours and opinions—all of which may also be rapidly evolving.

**Methods:**

We conducted in-depth interviews with 36 humanitarian staff involved in hygiene programme design in two crisis-affected settings—one a conflict affected setting (Iraq) and the other amid a cholera outbreak (Democratic Republic of the Congo). Interviews explored decision-making in each phase of the humanitarian project cycle and were thematically analysed.

**Results:**

Participants considered the design and implementation of hygiene programmes in crises to be sub-optimal. Humanitarians faced sector-specific challenges as well as more general constraints associated with operating within the humanitarian system. Programme-design decisions were made naturalistically and relied heavily on the intuitions and assumptions of senior staff. National organisations were often side-lined from programme design processes despite being in a better position to gather situational data. Consequently, programme design and decision-making processes adopted by humanitarians were similar across the two settings studied and led to similar types of hygiene promotion activities being delivered.

**Conclusion:**

Hygiene programming in crises-affected settings could be strengthened by initiatives targeted at supporting humanitarian staff during the pre-implementation programme design phase. This may include rapid assessment tools to better understand behavioural determinants in crisis-affected contexts; the use of a theory of change to inform the selection of programme activities; and funding mechanisms which encourage equitable partnerships, phased programming, regular adaptation and have programmatic components targeted at sustainability and sector capacity building. Initiatives aimed at sector reform should be cognisant of inter and intra-organisational dynamics, the ways that expertise is created and valued by the sector, and humanitarian habits and norms that arise in response to system constraints and pressures. These micro-organisational processes affect macro-level outcomes related to programme quality and acceptability and determine or limit the roles of national actors in programme design processes.

**Supplementary Information:**

The online version contains supplementary material available at 10.1186/s13031-022-00476-8.

## Introduction

Hygiene promotion is a critical part of humanitarian responses to crises and public health emergencies [1, 2]. Handwashing behaviour has the potential to curb the spread of diarrhoeal [3] and respiratory diseases [4–6] which are among the leading causes of mortality in the wake of crises [7].

However, designing effective hygiene programmes in crises or public health emergencies is a complex task. Literature reviews have identified major gaps in our understanding of what works to change and sustain hygiene behaviour in stable, non-emergency settings [8–12]. The majority of these reviews conclude that health information alone is unlikely to be sufficient to change behaviour. Evidence suggests that hygiene programmes must target a range of contextual barriers and enablers of behaviour (known as behavioural determinants)—including cognitive factors as well as factors in the social and physical environments that influence behaviour.

Evidence on the effectiveness of hygiene promotion during crises or outbreaks is even more sparce given the challenges of conducting research in these settings [13–15]. For example, little is known about the factors that may determine behaviour in these contexts [9] and behavioural and health outcome measurement has historically been poor [16]. In comparison to other components of water or sanitation programming in crisis-effected populations, hygiene behaviour change tends to be less well researched and resourced and is understood to require programmatic staff to have specialised capacities which are often lacking in crises [13, 17–20]. Humanitarians designing hygiene programmes during crises and outbreaks also face unique constraints. For example, humanitarian staff are typically under pressure to act rapidly and yet are expected to utilise evidence-based approaches [21–24]; to contextualise programmes despite having imperfect data on the local situation, behaviours and opinions [25, 26]; to regularly adapt approaches based on the dynamic and phased nature of crises, public discourses and community and stakeholder feedback; and to provide programming which is sensitive to the needs of vulnerable crisis-affected populations.

There have been some attempts to document the ways that humanitarians navigate this complex set of circumstances to design hygiene programmes in emergencies [19, 20]. In these studies, humanitarian actors explained that hygiene programming in crises primarily consisted of health education and ‘hardware’ (e.g. building handwashing facilities) or hygiene kit distributions. They also reflected that hygiene behaviour was rarely given operational priority, that there was a lack of familiarity with behaviour change approaches and how these could be applied to crises, and that there were barriers to assessing behavioural determinants and translating these into contextualised programming in a timely manner.

Within the humanitarian sector more broadly, research has explored the ways in which humanitarians make decisions under pressure and amid such uncertainty. Campbell and Knox [24, 27] summarised four types of decision-making approaches that are used in humanitarian crises. These include ‘classical/analytical decision-making’ which requires humanitarians to identify a range of programmatic options, appraise these, and select the option that is likely to work best given the circumstances. In contrast, ‘naturalistic decision-making’ [28] involves humanitarians relying on intuition and learned mental shortcuts to identify relevant courses of action. Alternative approaches include the ‘procedures and protocols’ approach which encourages decision-making to be guided by previously established standards and the ‘sensemaking’ approach [29] which requires humanitarians to iteratively identify patterns within the constantly changing state of information and adapt programming accordingly. All of these types of decision making may also be influenced by an individual’s self-interests, ideals, and preferences [30]. Campbell and Knox conclude that there are inherent strengths, limitations, biases, and feasibility constraints to applying each of these to humanitarian decision-making approaches and identify the need for further applied research to test their generalised findings [27].

Programme design and decision-making during humanitarian crises is also influenced by intra and inter-organisational power dynamics and the broader system of coordination and financing within the humanitarian aid sector. For example, in recent years there has been a strong push towards the localisation of aid through the Grand Bargain Commitments [31]; however donors, United Nations agencies, and international non-government organisations (INGOs) still dominate the sector in terms of financing and influence [32]. This greater influence enables certain types of programming norms to develop while limiting the participation and program design capabilities of national non-government organisations (NNGOs) or civil society actors [33]. To account for the influence of systemic and relational factors on decision-making, Heiss and Johnson outline a Unified Framework for Understanding International Nongovernmental Organizations. This highlights that actions taken by non-government organisations (NGOs) are influenced by ‘macro factors’ in the institutional environment and ‘meso factors’ related to the interactions between humanitarian actors, donors and nation states [30]. However, to date this framework has not been widely applied and has never been used to study the work of humanitarian actors.

This research set out to explore the ways that humanitarian actors involved in hygiene programme design, navigate the complexity of the humanitarian system, and imperfect states of evidence and contextual knowledge, to construct narrative accounts of what it is they do and why.

## Methods

This research is grounded in a constructivist research paradigm and explores the topic through two comparative case studies in Iraq and the Democratic Republic of the Congo (DRC). The research uses in depth interviews with humanitarians professionals who work in the water, sanitation and hygiene (WASH) sector and were involved in community-based hygiene programming.

### Study sites

We intentionally focused our work in two different types of crises, in geographically different regions and included different types of humanitarians. This allowed us to explore the influence of the context on programme design and how experiences of programme design differed between organisational types. In the Kurdistan Region of Iraq interviews were conducted between April and May of 2017 and related to the humanitarian response to the conflict between the Iraqi Government and their allies and the so-called ‘Islamic State’. Hygiene was key to mitigating diarrhoeal diseases among those displaced by the conflict who typically resided in densely populated camps where WASH facilities were shared. Interviews were also conducted in the Democratic Republic of the Congo (DRC) in October 2017. These interviews took place during the largest cholera outbreak in recent decades [34] and amid the broader complex crisis in the Eastern region of the country where there has been decades of conflict and displacement [35]. In this setting hand hygiene was considered key for interrupting cholera transmission [1, 36].

### Conceptual frameworks

In developing a conceptual framework for this research we utilised behaviour change and intervention design frameworks [37, 38] and the humanitarian programme cycle [39]. While the terminology and specific steps outlined in these frameworks and programme cycles differ, there are a lot of commonalities too [20]. For example, the Behaviour Centred Design (BCD) Framework and the Steps for Quality Intervention Development (6SQuID) outline similar steps to guide programme design. These processes include a problem exploration phase where the behaviour and target group are defined, and available literature is assessed to map what is already known. The second phase involves actively building on this state of information through contextual learning with the target population. The third phase typically involves translating the learning in the first two phases into intervention activities by identifying malleable factors and potential change mechanisms. The fourth phase involves making plans for the delivery of the programme including piloting potential activities on a small scale, training staff, and putting mechanisms in place to support iterative adaptation. The final phase in both approaches is to develop a plan for monitoring and evaluating the program (although this is not covered in this research). Prior research has acknowledged that this ‘ideal’ process of behaviour change programme design is challenging to implement in humanitarian crises and may not acknowledge all of the systemic constraints of working within these settings [20]. Therefore, in our work we choose to frame these intervention design steps within the humanitarian programme cycle which recognises additional aspects of programme design such as resource mobilisation and coordination, information management, capacity strengthen and sustainability. Table 1 below describes how these three frameworks were combined and defined within this research. These concepts and definitions informed the structure of the interview guides we developed.

**Table 1:** Definitions of the steps of humanitarian programme design as applied in this research

Phase of programme design	Detailed definition of each phase derived from the Behaviour Centred Design (BCD) Framework, the Steps for Quality Intervention Development (6SQuID) and the Humanitarian Programme Cycle
Developing programme proposals	Assessment of the population’s humanitarian needs in general and consideration of how to strategically respond to and prioritise activities which can meet needs in a coordinated fashion Problem exploration phase where available literature is assessed to map what is already known about behaviours, and ultimately define the specific behaviour and target group of the intervention Conduct additional contextual learning with the target population to address knowledge gaps Translate learning into intervention activities by identifying malleable factors and potential change mechanisms
Resource mobilisation	Secure funding to implement the humanitarian response programme and ensure proposed work is aligned to both donor requirements and to the work of other actors Ongoing negotiations and relationships with donors throughout programme design and implementation
Programme implementation and adaptation	Deliver the programme—including piloting the approach on a small scale, training staff, and putting mechanisms in place to support iterative adaptation
Coordination, information management, capacity and sustainability	Ongoing coordination between humanitarian actors working within the same region (both within and between sectors) with the aim of sharing learning, reducing duplication and maximising the efficiency of the response in being able to meet population needs Ongoing information management to support programmatic learning and share resources and insights that could strengthen programme quality Mapping of capacity gaps among humanitarian response actors and subsequently developing or identifying appropriate resources, trainings or capacity sharing opportunities to address these gaps Inclusion and implementation of initiatives which are designed to support recovery and resilience building, sustain programming, or transition programming into the hands of local actors

### Participant sampling

For this research the National WASH Clusters in Iraq and DRC served as our focal point for identifying research participants. The Humanitarian Cluster System was established in 2005 to address identified gaps in humanitarian action [40]. The WASH Cluster forms one of the 11 thematic coordination mechanisms typically established in the wake of a crisis and aims to strengthen the coordination and capacity of organisations working on WASH programming with the ultimate aim of improving the relevance, quality, coverage and effectiveness of interventions. In both study sites the WASH Clusters involved international non-government organisations (INGOs), local non-government organisations (NGOs), United Nations Agencies and government actors. The research was presented and explained to all actors at a WASH Cluster meeting and organisations were invited to identify the staff member/s who would be best placed to discuss the management, design and delivery of their organisation’s hygiene programming. In cases where the hygiene programming of an organisation could not adequately be summarised by one staff member, additional individuals were invited to participate. Follow up calls were made to organisations to identify suitable participants.

### Data collection

Interviews in Iraq were conducted in person by SW, who is of British ethnicity and has a background in behavioural science and WASH. Interviews in Iraq were generally conducted in English but in one instance SW was accompanied by an Arabic translator who provided simultaneous translation. Interviews in DRC were conducted in person by SW with simultaneous translation to either French or Swahili by ACM. ACM is Congolese and had prior experience working with NGOs as part of humanitarian programming. In both settings interviews typically took between 45 min and 1.5 h. Interviews were audio recorded, translated where necessary, and transcribed. Interviews continued until a point of saturation was reached or when all eligible organisations had been approached to participate.

### Data analysis

A preliminary analysis was conducted by taking interview notes, discussing these within research teams and validating findings through a participatory workshop in both countries. In Iraq the participatory workshop included 71 representatives from 31 different humanitarian agencies and in DRC the workshops involved 88 participants from 26 different agencies. In both cases preliminary findings were presented and feedback was sought on the contextual interpretation of findings.

A subsequent in-depth thematic analysis was led by SW and conducted based on the approach described by Braun and Clarke [41]. The coding frame was developed deductively and informed by the four phases of humanitarian behaviour change programme design that are described in Table 1. Framework matrices were developed for each code and themes were defined and described. These were validated by ACM and TH. A secondary parsing of the data related to programmatic decision-making was done by comparing findings to the frameworks outlined by Campbell and Knox [27] and Heiss and Johnson [30].

### Ethics and consent

Participants were informed about the study and that their opinions would be anonymised at an individual and organisational level. Written consent was provided by each participant. Ethics permission for the study was provided by the London School of Hygiene and Tropical Medicine (Protocol 13,545), the University of Kinshasa’s Public Health School (Approval no: 038/2017) and Hawler Medical University.

In Additional file 1 we also describe how our work adheres to the Standards of Reporting Qualitative Research [42].

## Results

A total of 24 interviews with 36 humanitarians were conducted, with 11 interviews taking place in Erbil and Dohuk in the Kurdistan Region of Iraq and 13 in Goma in Eastern DRC. The demographics of participants was consistent with the current state of senior staffing within humanitarian WASH response: the majority of participants were male (75%) and half were not nationals of the country where the crisis was occurring (50% foreign nationals). Similarly, the participating organisations reflected the composition of the WASH Clusters, with INGOs being the most common type of participant organisation (57%). The majority of people interviewed held WASH Programme Manager roles. A detailed description of characteristics is provided in Table 2.

**Table 2:** Summary of the characteristics of interview participants

*Number of organisations (n = 24)*	
Iraq	11
DRC	13
Number of organisations interviewed in both countries	5
*Number of humanitarians participating in interviews (n = 36)*	
Iraq	17
DRC	19
*Gender of participants (n = 36)*	
Male	27
Female	9
*Nationality (n = 36)*	
Congolese	12
Iraqi/Kurdish	6
Foreign nationals	18
*Types of organisations participating (n = 21)*	
International Non-Government Organisations	12
National Non-Government Organisations	5
UN Agencies	3
Government	1

### Developing programme proposals

The process for developing the hygiene component of WASH proposals was described as ad-hoc and constrained by tight submission deadlines. Humanitarians recognised that the processes they used were sub-optimal but faced frustration and challenges in trying to operate differently:

*“Sometimes when you are doing this work [programme design], you feel like you are a guinea pig stuck on a wheel. You can see what you want to do, what the right thing to do is, but for one reason or another you can’t get there”.* (INGO, DRC).

When designing hygiene promotion activities, participants explained that there was an over-reliance on the prior experiences and expertise of senior WASH staff, with limited contributions from frontline staff:

*“So this is one of the weaknesses…With our organization [proposal writing] basically stops at the program manager level in terms of technical expertise…and so everything we do in terms of WASH is our own, not related to the organization, so there is no institutional documents or strategies or ways forward, so that is inherently kind of risky and short lived, because it can’t last longer than the people do in the place.”* (INGO, Iraq).

If individuals required additional resources or information to support assessments or the development of hygiene promotion activities, most turned to resource collections that they had personally acquired over the years or used online search engines to find relevant materials:

*“It is a bit of kind of feeling your way through. I mean this is why Google is a great thing to go and find documents and the support you might need because everything is there. It is better than just relying on one like theory or methodology or approach…But again…I wouldn’t say this is good programming.”* – (INGO, Iraq).

Many participants explained that their organisations did produce a range of resources to guide programming but that these were often not user friendly. Participants admitted that behavioural theory was rarely used to inform programming, partly because there were “*so many books, so many approaches*” and that these were “*text heavy*” making it hard to find the information required. Organisational guidelines did seem to inform the overarching principles of a programme proposal. For example, certain organisations had preferred delivery mechanisms (e.g. setting up care groups [43]) or inclusivity principles (e.g. a focus on gender equity).

Most participants reported using standard assessment tools that were either used throughout their organisation’s global programmes or standardised by the National WASH Cluster. These tools tended to be multi-sectoral and designed to prioritise humanitarian needs. If hygiene behaviours were specifically explored this was typically done through Knowledge, Attitude and Practices (KAP) surveys. Some organisations complemented this with key informant interviews or focus group discussions. These behavioural assessment tools were considered time consuming and required a certain level of staff experience. Many organisations explained that they were not able to always conduct behavioural assessments prior to developing programme proposals:

*“I would say no we don’t do it [behavioural assessment] before the proposal. It’s normally because… without having personnel and unrestricted funds to do it, like a KAP survey is not cheap because you have to have daily workers…you have to have the tablets available, you have to do the analysis, it’s not quick and it’s not easy.” – INGO, Iraq*.

Participants reported that KAP surveys and other common methods for understanding behaviour predominantly focused on access to products and infrastructure, handwashing knowledge, and reported practice. Available tools were less able to provide a more nuanced understanding of the determinants of hygiene behaviour in a particular context. Several participants acknowledged that populations typically understood the health benefits of handwashing, but that there was a gap between ‘knowing and doing’. When asked about the determinants of handwashing behaviour in their context many participants indicated that this was the remit of experts or specialist researchers who were not feasible to engage in crises.

Given that organisations were often unable to conduct rapid assessments prior to proposal submission, many indicated that for the hygiene component of their programmes they had *“learned to be a bit vague in proposals on purpose”*. In such cases the programmatic scope of work and budget tended to be based on standardised approaches and materials, such as materials or guidance created by Global or National WASH Clusters, and then organisations would commit that these would be modified and contextualised over the course of the response as necessary.

National staff members within INGOs or NNGOs tended to be in a stronger position to get real-time information from communities or to make ‘informed assumptions’ that could guide programme design based on their prior experiences of working within the context. During interviews national staff members appeared to identify with crisis-affected populations more directly. However, some national staff members were also more likely to form stereotypical judgements about the behaviours or attitudes of crisis-affected populations if they came from cultural groups or circumstances that were different to their own:

*“We have like different levels of people. You have like the ‘top level’ and of course they are educated. If you go to them and you tell them about hand washing then maybe they are going to welcome you…So I think to start with them is good, as their mentality is already better than the poor people. The poor people will just say ‘oh come on I’m living in a terrible situation and you are coming here wanting to talk to me about hygiene.’”* (NNGO, Iraq).

*“The problem is that the cholera outbreak can be affected by the culture, because we can sensitize people, but others remain unchangeable… We can tell them to wash hands, but it is all about their mindset. We ask them to leave that kind of culture that our grandparents used to practice behind*” (NNGO, DRC).

Hygiene promotion initiatives were rarely standalone programmes but rather were integrated into broader WASH or disease control programmes. However, in both settings, hygiene promotion activities were, perhaps justifiably, considered to be less of a priority than other WASH components, for different context-specific reasons. In Iraq this was because humanitarians felt that the population typically had high rates of handwashing behaviour prior to the crisis and that therefore the priority was to restore damaged water systems to facilitate these behaviours again. In DRC most humanitarians felt that *“cholera is water”* meaning that contaminated water reservoirs and water scarcity were the primary factor contributing to both transmission and limited handwashing practices. Some participants also explained that the prioritisation of water and sanitation infrastructure in proposals was because the *“technical side is the easy part”* and because it is more costly*.*

### Resource mobilisation

Given that donors hold funding and shape funding calls and timelines, they were recognised to have substantial indirect influence on the content and quality of programming:

*“The donors heavily influence our strategy in the sense that there is never enough money, so we have to kind of answer to them a bit. Unfortunately, we are not in a very good negotiating position yet to turn around and say ‘no we don’t want to do this’, or to refuse money.”* (INGO, Iraq).

*“The funny thing about this emergency side of things is that often it is the grants and the donors that are the time constraint rather than the actual emergency. They could be the key to forming a good program... but they don’t allow time to actually sit and plan out a good intervention.”* (INGO, Iraq).

Participants generally felt that hygiene was underfunded in relation to other aspects of WASH and explained that this was because organisations and donors typically underestimated the cost of doing hygiene promotion well:

*“One thing that is really important to me is to push people to include more budget for hygiene promotion. Because they [donors] want us to do a lot of things regarding hygiene promotion, huge targets, but all I have is a team of 9 persons and $5000 USD for the whole year. If you want to do nice things, or innovative things then it needs to be properly taken into account in the budget – it’s an often forgotten area.”* (INGO, Iraq).

Others explained that when donors asked them to reduce the budget of their WASH programme, hygiene was typically where financial cuts were made.

Hygiene programming was also affected by broader patterns in humanitarian funding. For example, participants remarked that humanitarian funding often came all at once or not at all, as it was so closely tied to the initiation of a crisis event or donor perception of the severity of the crisis. In Iraq people mentioned that the ‘*humanitarian circus’* quickly moved from one conflict to the next in a way that rarely mirrored the needs of the population. In DRC, multiple participants described receiving emergency funding for short-term soap distributions or water chlorination programmes during the peak of the cholera outbreak but felt that the money would have been better spent on building safe and effective water systems to prevent the next outbreak. Some organisations had started to exploit patterns in emergency funding by framing all their work within an ‘emergency’ discourse, even though cholera outbreaks in DRC are relatively predictable (i.e. they happen annually):

*“You find only funding for emergency, so everybody is putting this in their presentations and everyone’s communicating saying it’s an ‘emergency’, because this is how you get funds.”* (INGO, DRC).

Most NNGOs reported that they rarely received funding directly from bilateral or multilateral donors, but rather via UN Agencies or INGO partners who sub-contracted a lot of the hygiene activities to them. Commonly they felt that this was because international actors didn’t trust their financial management or technical skills. This meant that NNGO actors were often unable to be as responsive as they could be at the outset of a crisis. The unpredictability of finances also made it hard for them to undertake transitional or development work:

“*Most international organizations intervene in emergences only. It is a problem, they just come when there is an emergency and they say we are there ready to support you. But we are a national society, here all the time, and when there is no emergency, we cannot see any help*” (NNGO, DRC).

A representative from the Government in DRC expressed frustration at the funding of the humanitarian system, explaining that they had hoped that the establishment of a National Cholera Roadmap would make funding around hygiene more aligned to Government plans. However humanitarian actors continued to secure funding directly with donors and often only came to the Government when grants had been awarded. With a lack of Government funds to support hygiene, the Government often just agreed to whatever organisations proposed—a situation that the participant compared to being “*like a lion if it is hungry—at that point we take what we can get*”.

### Programme implementation and adaptation

There was a relatively high level of consistency in the types of interventions delivered across countries and organisations. When asked about specific activities, participants typically described the delivery modalities rather than the content of their programming. Participants explained that hygiene interventions commonly included household-level visits, community meetings, the development of posters or other communication materials, collaboration with women’s groups, or the distribution of hygiene kits. However, when asked what happened at household visits, for example, descriptions were more vague, with participants just saying that their staff ‘*sensitised*’ or ‘*mobilised*’ community members to adopt handwashing practice. None of the participants were able to articulate a theory of change for how they planned to influence hygiene behaviour.

Among INGO staff who had experience working across multiple crisis-affected contexts, there was a belief that hygiene programmes were rarely innovative. One participant explained that innovation is curtailed by the nature of crises which don’t lend themselves to programmatic risk-taking:

*“People are so worried about the potential risk of varying from these traditional approaches because they think they are just so involved in the business of saving lives that they don’t have time to do any things better and more creatively, even if that might actually save more lives!...But I think part of the job is convincing and sensitising people within the sector that actually we can do something much more fine-tuned to improve hygiene programs. It doesn’t require reinventing the wheel but just taking time to understand.” (INGO, Iraq)*.

Several participants felt that hygiene programmes were likely to be more effective if programmes were of a longer duration and if frontline staff regularly engaged with communities so that they could build meaningful relationships. At the same time others cautioned that just repeating messages through the same modality is likely to cause crisis-affected populations to disengage from hygiene promotion programmes:

“*If it’s just a one-off, first of all, you won’t receive the impact so it is hard to measure, but if people know that we are coming back time to time as you follow up…then we act as like as social workers, we are not just NGO guys who distribute stuff, they will talk to us, know us by name and will be very friendly. Then you kind of lose this barrier of humanitarian worker and IDP, you become more similar.*” (INGO in Iraq).

*“You have to change the way that you are transferring the message…I mean it’s not really nice to go and make tent to tent visits on a daily basis, you shouldn’t have to bother them, you have to find a new methodology, you have to make it something nice for the people. Otherwise if you are not doing a good program I’m sure they will get bored and they will tell you 'please we’ve heard a lot and we know how to practice, you just continue to teach us'.”* (NNGO in Iraq).

Community engagement was mentioned frequently by participants as something that should happen throughout hygiene programming. However, there were inconsistent conceptualisations of what community engagement should be. For some, community engagement was primarily something that was considered at the assessment stage, for others it meant working in close collaboration with local governments or civil society organisations. Many actors suggested that community engagement was designed to encourage community ownership in relation to hygiene behaviour and the management of handwashing facilities. This was seen as important because of the short duration of most emergency hygiene programmes:

*“We are preparing the community to take charge because we know that a day would come when the project will stop... So, only working with [INGO] staff while we know that one day [our organisation] will close its offices…could not be wise.” (INGO, DRC).*

Some organisations explained that community ownership was built through repeated trainings while others designed their programmes in such a way that there was an expectation that crisis-affected populations would be willing to ‘volunteer’ to share hygiene promotion messages or to be part of ‘village committees’ which would be involved in building or overseeing operation and maintenance of handwashing facilities. Some organisations paid community members small stipends for this work while others did not.

The majority of the participating organisations indicated that there were no formal processes informing programmatic adaptation and contextualisation. Participants explained that contextualisation typically involved translating generic communication materials into local languages, adjusting images so that they looked more like people in the communities where they were working, changing the delivery channels or adjusting the contents of hygiene kits to include locally acceptable products. One participant explained that this type of contextualisation of hygiene programmes was too superficial:

*“You have seen the [standard] tools which are made for hygiene promotion, they are a package, but to be honest they should always be adapted to a context. I have seen those tools replicated for the last 4 years in all the places…I once spent 3 days with other WASH fellows revising them but it was too much ‘money for nothing’, just to say oh the colour is not good, the hat people wear here is different… It is more important to really go in deep with communities and understand what is working or not – not just adapting those hygiene promotion tools for the sake of adapting the tools.”* (INGO, Iraq).

Programmatic adaptation relied heavily on the prior experiences of WASH staff or the views of implementation staff about the communities where they worked. However, biases within these personal perspectives could sometimes compromise programme decision making:

*“When I came in, I had African-based views of what hygiene promotion should look like .... And this is not Africa and so I think people are kind of offended. I have heard these kind of comments from them – ‘this is a rich country we don’t need anyone to come and, you know, do these kinds of approaches’.”* (INGO, Iraq).

The ability for programme implementers to adapt and address changing needs was often contingent on relationships with donors, the duration of the project, organisational priorities and the capacities of frontline staff:

*“I think it depends on the time you have to implement your project. If you have a 1 year project then normally the donors are kind of flexible so you can kind of adapt your project…But if you have a 3 month project which is what normally happens in emergency areas, at most 6 months, then it is difficult to adapt.”* (INGO, Iraq).

Longer-term programmes, which did exist in DRC, were more able to conduct thorough initial assessments (post the grant being awarded), develop constructive two-way communication with donors and adapt to changing circumstances:

*“For our [long-term project] we are saying that the project started in March, but the month of March and the month of April were only oriented towards doing the assessments and you understand that the assessments are accompanied by a report and the report that will be discussed with the donor, and all that follows is a discussion about how the programme will be designed to reflect the things we have learned.”* (INGO, DRC).

*“If you have an intervention which is an ongoing intervention with competent trained staff, then there is no problem, you can react to a new cholera outbreak, for example. And it is likely that the quality and the speed of the interventions will be much better.”* (INGO, DRC).

Participants in both countries explained that hygiene programmes were often curtailed by security issues, which delayed humanitarian staff from gaining access to populations. Population movement was cited as a challenge in both countries. In DRC this was because target populations were often only displaced temporarily while others had been IDPs for many years. In Iraq the inability for some IDPs to leave camps limited the programming options available to staff. For example, they had to distribute hygiene items rather than use cash or voucher systems to facilitate access through markets. Finally, perceptions towards displaced people by government authorities were raised as programmatic challenges in both countries, with humanitarians explaining that there was sometimes resistance towards providing high quality hygiene or WASH infrastructure to populations as authorities felt this would discourage people from returning home.

### Coordination, information management, capacity and sustainability

Generally, participants viewed coordination platforms (like the national and sub-national WASH clusters) positively and felt that this had led to the gradual improvement of programmatic quality and the alignment of humanitarian responses. Specifically, people thought that the cluster system played a key role in mapping what actors were doing and where, minimising duplication, resolving common challenges, mobilising resources, and promoting regular communication between organisations involved in hygiene promotion. Factors that contributed to the success of coordination platforms included the involvement of government authorities, an agreed hygiene or WASH plan for organisations to align their work to, and the skills of the person leading the coordination mechanism.

Coordination challenges related to harmonisation, participation, and sustainability. Some participants explained that coordination platforms encouraged an over-reliance on standardised hygiene approaches. While these individuals saw value in the harmonisation of hygiene messages and activities across organisations, lengthy central approval processes often delayed action and curtailed innovation and contextual adaptation within programmes. Other participants explained that coordination was often limited by the fact that some response actors did not regularly participate, share their programmatic information or contribute to joint decision making. Larger INGOs were often seen to “*do whatever they want*” because of their financing and programmatic influence.

Some participants felt that the establishment of WASH clusters had the potential to contribute to response programmes which were built upon prior collective learning. However, mechanisms to support knowledge management between actors were often lacking and hampered by high levels of staff turn-over. This commonly resulted in a short-term institutional memory loss.

*“Like the WASH cluster has been active since 2014 so they must have some collective experience…but it’s quite vague on where to find this.”* (INGO in Iraq)

Some participants explained that larger INGOs were in a stronger position to support sector learning since there are often staff at a headquarters level responsible for knowledge management and sharing lessons learned from previous projects.

Participants highlighted that there was often a skills gap around hygiene programming. Some people explained that this may be because the WASH sector has historically been dominated by engineers whose training and interest in doing ‘soft’ hygiene promotion programming is likely to be limited:

*“I think that we have very many well qualified WASH staff but the vast majority of them are qualified as engineers and I think trying to get them to understand about hygiene is complicated, they don’t really see it as important. This is why hygiene had been side-lined for so long.”* (INGO in Iraq).

The majority of hygiene promotion staff currently develop their skills on the job. However, many organisations reported that humanitarian crises are not an ideal learning environment, and that meaningful capacity building is not possible due to the short duration of programmes. Several participants suggested that the skills required for hygiene promotion are hard to teach and that hygiene promotion requires people with a certain type of personality to make programmes successful:

*“I think hygiene promotion needs creativity because when you design a session, you need some people with charisma or kind of leadership. Those are real skills, but it is not something you can learn from the book. If you find those people, yeah, it’s very important to keep them.”* (INGO in Iraq).

Others explained that there are few training programmes for hygiene promoters and no recognised qualifications or pathway into the sector.

*“It’s a funny, bizarre sector, people dip in and dip out and come from all different backgrounds and some people invest in themselves to get into the sector and some people just kind of swing by and then move on to something else… If you want hygiene promotors who really understand the purpose of the job then there needs to be some sort of investment in the sector in the human resources side….we need to improve the overall professionalization of hygiene so that people treat it as a career.”* (INGO in Iraq).

Representatives from NNGOs were more likely to report skills gaps. This was because they were often tasked with conducting the bulk of hygiene promotion activities when working in partnership with larger INGOs but often felt ill equipped to carry this out:

*“We tell them that we will need like an expert to advise us and do what is required. Because it’s like, we are not academic people, we didn’t study hygiene promotion and it requires a good deal of experience... Instead, they leave the behaviour part to us as the local NGOs and that is why a lot of NGOs are just transferring messages tent to tent. But people have trauma and it’s not correct to just put some promotors in a camp and get them to say wash your hands - no!”* (NNGO in Iraq).

In thinking about sustainability, participants explained that hygiene programming in crises had to be thought of in phases and that sustainable solutions were not feasible to consider in the acute stage of the response. However, many recognised that sustained or sufficient funding beyond the acute phase of the crisis was rare, meaning that, practically, few sustainable actions could be considered. The sustainability of hygiene programming emerged as a greater concern in DRC given that short-term hygiene promotion initiatives had been going on for 25 years since the first cholera outbreak in 1994:

*“The particularity of our country is that we are in a situation where emergencies do not end… it is well known that every year during the dry season there are always problems of cholera…and yet each time that there are cases the humanitarian community mobilizes… It should be a chain, so we start from the emergency, and then there follows a transition for early recovery, and then we could go now for development, but our context does not allow it… then the biggest problem is that there are perhaps structural causes that should not normally be part of the humanitarian mandate but should be regulated by the authorities.” (UN Agency, DRC).*

Several organisations in DRC explained that they had set up emergency and development teams within their structures to better bridge this divide. One organisation mentioned that they had focused on building durable handwashing infrastructure in the hope that this would have lasting benefits beyond the programme. In both Iraq and DRC most participants expressed a desire to align their work more closely with government or other sustainable community structures. This remained a priority even though many people described how challenging this was or that intensive efforts to do this to date had had limited success. Some actors had greater success when building relationships with district level government representatives rather than provincial or national as they were more aware of localised concerns.

## Discussion

**Fig. 1:**
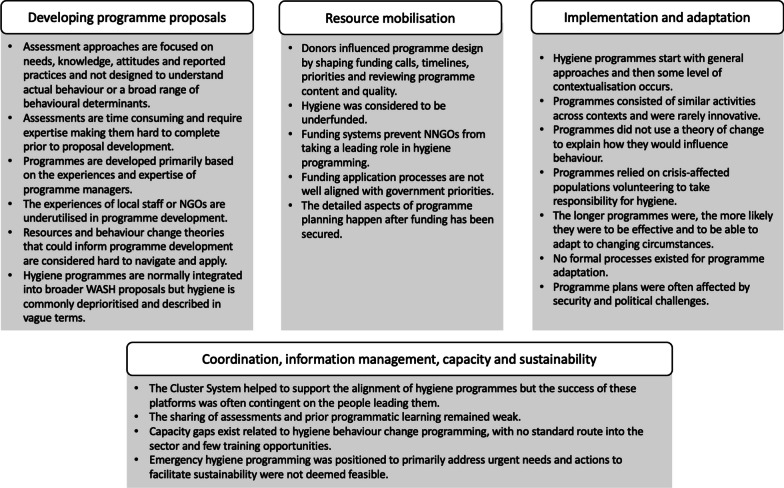
Research findings mapped across the phases of programme design and delivery

Participants in our study were self-reflective about the work of their organisations and openly critiqued common approaches to hygiene promotion and behaviour change in the WASH sector. It was clear from our interviews that humanitarian participants cared about the populations they served and aspired to implement hygiene programmes that were consistent with sector guidelines, engaged communities in participatory programming, strengthened local capacities and community ownership, and operated in collaboration with government and other response partners. Many were also aware of more ‘ideal’ or systematic processes of programme design but in practice, struggled to apply these processes or behavioural theories to crisis-affected settings. This led to frustration among humanitarian staff and programmes that were perceived to have a limited impact on behaviour. Figure 1 provides a summary of the research findings across each of the stages of programme design and delivery. These findings are generally consistent with prior work on this topic by Vujcic et al. [19] and Czerniewska and White [20].

### The importance of the pre-implementation design phase of programming

While many aspects of systematic programme design processes are compromised in humanitarian settings, our work identified that the pre-implementation, design-focused phase of programming (which may range from a matter of days to about a month) is the period which has the potential to most substantially shape the content and quality of programming. Our findings suggest that this programme design phase and the process of proposal development could be strengthened by the following types of initiatives:

- Developing rapid assessment tools which explore a broad array of hygiene behavioural determinants and then developing staff capacities to utilise these tools so that they can inform programming.
- Developing processes which make it easier for humanitarians to analyse behavioural assessment data (particularly qualitative data) and translate these into contextualised program design strategies.
- Effectively communicating behavioural theory or evidence-based hygiene promotion approaches to humanitarians in such a way that this information can be accessed, navigated, adapted, and applied in crises.
- Developing processes which facilitate the involvement of multiple actors in proposal development and promote equal and transparent partnerships between donors, UN agencies, INGOs, and NNGOs.
- Establishing funding mechanisms which encourage phased, adaptive, and sustainable programming.
- Research and applied tools which allow humanitarians to better estimate the realistic costs of effective hygiene programming.
- Capacity mapping and strengthening related to hygiene behaviour change.

### Consistency of findings across study settings

Despite DRC and Iraq experiencing different types of crises, and being different geographical and cultural contexts, the constraints humanitarians faced when designing programmes, and the way they made decisions within these settings, were remarkably similar. The challenges reported may reflect the broader constraints of working within the humanitarian system and may therefore be true of other response initiatives, including other aspects of humanitarian WASH programming or other behavioural interventions in crisis-affected settings [27]. The similarities between processes and decision-making in both contexts may also explain why the nature of hygiene programming in both locations was similar—with a heavy focus on health education and the distribution of hygiene products and infrastructure. Knowledge about handwashing and the creation of an enabling environment are likely to be key to facilitating behaviour change but these components alone are likely to be insufficient [8, 44]. Across both countries there were commonalities in the discourse and framings chosen by participants which serve to perpetuate certain types of action. Implicitly, hygiene programming (alongside other aspects of WASH programming) was constructed by those within the sector as an inherently good public health interventions that could be implemented without detailed engagement with broader socio-political realities, and with minimal concern for unintended consequences of programmatic decisions on crisis-affected populations or the state.

### Decision-making and the power dynamics that affect programming

The majority of decision-making related to programme design occurred at the micro-level and was influenced by the internal hierarchies of humanitarian organisations. Specifically, nationally-based, foreign WASH coordinators appear to be the dominant force in shaping hygiene related programmatic decisions. Programme proposals are developed with little input from other local or regional stakeholders. Many of these individuals in our study recognised that their approaches to hygiene programme decision-making were biased and were not as evidence-based or contextualised as they would have liked, but felt that more consultative or analytical decision-making was not feasible in crises. As such the majority of hygiene-related programmatic decisions are currently being made using a ‘naturalistic approach’ which draws on the ‘embodied tacit knowledge’ of these senior WASH staff [45, 46]. An over-reliance on individual tacit knowledge has been acknowledged as a widespread challenge in the humanitarian sector due to high levels of staff turn-over, a tendency to approach every crisis as unique, and weak accountability mechanisms associated with fragmented humanitarian power structures [46–48]. Tacit knowledge may be held by both individuals and organisations, but when programmes are designed primarily by senior WASH staff opportunities for organisational or sector-wide learning and change are likely to be missed.

‘Meso-level’ and ‘macro-level’ factors also shaped the nature of hygiene programming in these crisis-affected settings and perpetuated this culture of self-reliance among senior WASH staff while also narrowing the scope of how ‘expertise’ is valued and constructed within the sector. For example, cultural norms within the sector meant that when senior staff identified gaps in their expertise, they were more likely to google solutions or look ‘up the hierarchy’ towards senior organisational experts, rather than exploring opportunities to fill knowledge gaps through research among crisis-affected populations or through the engagement of national staff in the programme design process. These ‘ways of knowing’ prioritise the diffusion of technical expertise from powerholders in the ‘Global North’ and allow inequitable power dynamics between foreign and national staff and between INGOs and NNGOs to persist [49–51].

Our findings also indicated that NNGOs were well positioned to undertake rapid assessments, shape hygiene proposal development, and implement programmes. However, inherent biases within the humanitarian environment such as the lack of sustained funding, the demands of funding calls, and the assumption that capacity strengthening is beyond the scope of humanitarian programming, prevented NNGOs from maximising their potential. Furthermore, relationships between humanitarian actors, and the power dynamics between individual organisations and their donors, created barriers for NNGOs to secure funding (e.g. donors didn’t trust the financial and programmatic capacity of NNGOs) or to negotiate for more flexible, contextualised and sustained programming. While some of these challenges have been identified in other sectors [52, 53], our findings suggest that the hygiene sector needs substantial reform to realise the Grand Bargain Commitments which aim to improve the effectiveness and efficiency of humanitarian aid through localisation and investment in capacity strengthening at national levels [31]. The findings of our research indicate that effective reform must pay attention to inter and intra-organisational dynamics, decision-making and knowledge creation because these micro-organisational processes affect macro-level outcomes and determine or limit the roles of national actors in programme design processes [51].

### Limitations

Our findings represent the opinions and experiences of humanitarians in just two specific settings and therefore may not be transferrable to all types of crises or across other diverse geographies. Certain relevant voices were also not fully represented in this research. For example, government actors involved in humanitarian response were contacted to be part of this research in both countries, but only one individual in DRC was able to participate. Given varying engagement in coordination mechanisms, future similar research should consider having a separate process for identifying government stakeholders. Our work could have also been strengthened by including the voices of WASH donors, given their evident influence on hygiene programme design. Understanding the quality, acceptability and effectiveness of hygiene programmes in emergencies should also foreground the views of crisis-affected populations. While not reported here, we conducted complementary in-depth qualitative research with affected populations in both settings [54, 55]. Finally, 75% of our participants were male across the two countries and while this reflected the demographics of the sector, the voices and opinions of female WASH staff are under-represented in this work and merits further exploration.

As mentioned, the first author (SW) who is an academic of British origin, led the interview process in both countries and conducted the analysis. Her ‘outsider’ status [56] may have affected the way that participants responded to questions and the way results were interpreted given that she was external to both the humanitarian sector and the research locations. This positioning may have also allowed the participants to be more open with their responses [57]. To mitigate the potential biases that this may have brought, research notes were taken daily and preliminary research findings were shared with humanitarian actors at global and national levels.

## Conclusion

We found that WASH programme staff faced sector-specific challenges as well as more general constraints associated with operating within the humanitarian system. Consequently, the programme design and decision-making processes adopted by humanitarians in our study were similar across the two settings studied and led to similar types of hygiene promotion activities being delivered. Hygiene behaviour change requires an understanding of the contextual determinants of behaviour, the use of theory and evidence to inform locally relevant hygiene promotion activities, regular adaptation and intentional efforts to support sustainability. However, the humanitarian imperative to act rapidly [58–60] undermines the ability for any of these steps to be carried out effectively. Thus, while hygiene programmes in stable settings are increasingly making use of evidence and theory and designing contextualised programmes which are responsive to local circumstances, the humanitarian sector have been struggling to replicate these developments. Improving hygiene programming in crisis-affected settings will require a re-imagining of standard programme design processes so that they can be utilised within the constraints of the humanitarian system. Improved practice will also require a heightened awareness of the habits and norms that have emerged among humanitarians in order to deal with system constraints and time pressures. These unquestioned patterns of behaviour and the standard discourse around programme design may have detrimental effects on programme quality and cause unintended consequences to crisis-affected populations.

### Supplementary Information

**Additional file 1:** Alignment with the Standards for Reporting Qualitative Research.

## Data Availability

The datasets generated during the current study are not publicly available because even with redaction some deductive disclosure is likely. The dataset is available from the corresponding author on reasonable request.

## References

[1] D’Mello-Guyett L, Gallandat K, Van den Bergh R, Taylor D, Bulit G, Legros D (2020). Prevention and control of cholera with household and community water, sanitation and hygiene (WASH) interventions: a scoping review of current international guidelines. PLoS ONE.

[2] Sphere Association. The sphere handbook: humanitarian charter and minimum standards in humanitarian response. Geneva; 2018.

[3] Wolf J, Hunter PR, Freeman MC, Cumming O, Clasen T, Bartram J (2018). Impact of drinking water, sanitation and handwashing with soap on childhood diarrhoeal disease: updated meta-analysis and meta-regression. Trop Med Int Health.

[4] Aiello AE, Coulborn RM, Perez V, Larson EL (2008). Effect of hand hygiene on infectious disease risk in the community setting: a meta-analysis. Am J Public Health.

[5] Jefferson T, Del Mar CB, Dooley L, Ferroni E, Al-Ansary LA, Bawazeer GA, van Driel ML, Jones MA, Thorning S, Beller EM, Clark J, Hoffmann TC, Glasziou PP, Conly JM (2020). Physical interventions to interrupt or reduce the spread of respiratory viruses. Cochrane Database of Syst Rev.

[6] Moncion K, Young K, Tunis M, Rempel S, Stirling R, Zhao L (2019). Effectiveness of hand hygiene practices in preventing influenza virus infection in the community setting: a systematic review. Can Commun Dis Rep.

[7] Connolly M, Gayer M, Ryan MJ, Salama P, Spiegel P, Heymann DL (2004). Communicable diseases in complex emergencies: impact and challenges. The Lancet.

[8] De Buck E, Van Remoortel H, Hannes K, Govender T, Naidoo S, Avau B (2017). Approaches to promote handwashing and sanitation behaviour change in low-and middle income countries: a mixed method systematic review. Campbell Syst Rev.

[9] White S, Thorseth AH, Dreibelbis R, Curtis V (2020). The determinants of handwashing behaviour in domestic settings: an integrative systematic review. Int J Hyg Environ Health.

[10] Watson J, Cumming O, MacDougall A, Czerniewska A, Dreibelbis R (2021). Effectiveness of behaviour change techniques used in hand hygiene interventions targeting older children – a systematic review. Soc Sci Med.

[11] Wilson S, Jacob CJ, Powell D (2011). Behavior-change interventions to improve hand-hygiene practice: a review of alternatives to education. Crit Public Health.

[12] Martin NA, Hulland KRS, Dreibelbis R, Sultana F, Winch PJ (2018). Sustained adoption of water, sanitation and hygiene interventions: systematic review. Trop Med Int Health.

[13] Ramesh A, Blanchet K, Ensink JH, Roberts B (2015). Evidence on the effectiveness of water, sanitation, and hygiene (WASH) interventions on health outcomes in humanitarian crises: a systematic review. PLoS ONE.

[14] Taylor DL, Kahawita TM, Cairncross S, Ensink JHJ (2015). The impact of water, sanitation and hygiene interventions to control cholera: a systematic review. PLoS ONE.

[15] Lantagne D, Lehmann L, Yates T, Gallandat K, Sikder M, Domini M, String G (2021). Lessons learned from conducting six multi-country mixed-methods effectiveness research studies on water, sanitation, and hygiene (WASH) interventions in humanitarian response. BMC Public Health.

[16] Blanchet K, Ramesh A, Frison S, Warren E, Hossain M, Smith J, Knight A, Post N, Lewis C, Woodward A, Dahab M, Ruby A, Sistenich V, Pantuliano S, Roberts B (2017). Evidence on public health interventions in humanitarian crises. The Lancet.

[17] D’Mello-Guyett L, Yates T, Bastable A, Dahab M, Deola C, Dorea C (2018). Setting priorities for humanitarian water, sanitation and hygiene research: a meeting report. Confl Heal.

[18] World Health Organization (2020). Hygiene: UN-Water GLAAS findings on national policies, plans, targets and finance.

[19] Vujcic J, Ram PK, Blum LS (2015). Handwashing promotion in humanitarian emergencies: strategies and challenges according to experts. J Water Sanit Hyg Dev.

[20] Czerniewska A, White S (2020). Hygiene programming during outbreaks: a qualitative case study of the humanitarian response during the Ebola outbreak in Liberia. BMC Public Health.

[21] Bradt D (2009). Network paper: evidence-based decision-making in humanitarian assistance.

[22] Dijkzeul D, Hilhorst D, Walker P (2013). Introduction: evidence-based action in humanitarian crises. Disasters.

[23] Müller-Stewens G, Dinh T, Hartmann B, Eppler MJ, Bünzli F, Müller-Stewens G, Dinh T, Hartmann B, Eppler MJ, Bünzli F (2019). Humanitarian organizations under pressure. The professionalization of humanitarian organizations: the art of balancing multiple stakeholder interests at the ICRC.

[24] Campbell L, Knox CP (2018). Making Operational Decisions in Humanitarian Response: A Literature Review.

[25] Colombo S, Pavignani E (2017). Recurrent failings of medical humanitarianism: Intractable, ignored, or just exaggerated?. The Lancet.

[26] Colombo S, Checchi F (2018). Decision-making in humanitarian crises: politics, and not only evidence, is the problem. Epidemiol Prev.

[27] Knox Clarke P, Campbell L (2020). Decision-making at the sharp end: a survey of literature related to decision-making in humanitarian contexts. J Int Humanitarian Action.

[28] Lipshitz R, Klein G, Orasanu J, Salas E (2001). Taking stock of naturalistic decision making. J Behav Decis Mak.

[29] Weick KE, Sutcliffe KM, Obstfeld D (2005). Organizing and the process of sensemaking. Organ Sci.

[30] Heiss A, Johnson T (2016). Internal, interactive, and institutional factors: A unified framework for understanding international nongovernmental organizations.

[31] Inter-Agency Standing Committee. The Grand Bargain - Workstreatm 2: Localisation - More support and funding tools for local and national responders. : Inter-Agency Standing Committee, 2018.

[32] Roepstorff K (2020). A call for critical reflection on the localisation agenda in humanitarian action. Third World Q.

[33] Contu A, Girei E (2013). NGOs management and the value of ‘partnerships’ for equality in international development: What’s in a name?. Human Relat.

[34] Ingelbeen B, Hendrickx D, Miwanda B, van der Sande MAB, Mossoko M, Vochten H (2019). Recurrent cholera outbreaks, Democratic Republic of the Congo, 2008–2017. Emerg Infect Dis.

[35] Refugee Studies Centre (2010). Dynamics of conflict and forced migration in the Democratic Republic of Congo: experts workshop: report.

[36] Wolfe M, Kaur M, Yates T, Woodin M, Lantagne D (2018). A systematic review and meta-analysis of the association between water, sanitation, and hygiene exposures and cholera in case-control studies. Am J Trop Med Hyg.

[37] Wight D, Wimbush E, Jepson R, Doi L (2015). Six steps in quality intervention development (6SQuID). J Epidemiol Commun Health.

[38] Aunger R, Curtis V (2016). Behaviour centred design: towards an applied science of behaviour change. Health Psychol Rev.

[39] OCHA. Humanitarian Programme Cycle Humanitarian Response2020 [Available from: https://www.humanitarianresponse.info/en/programme-cycle/space.

[40] Inter-Agency Standing Committee. Guideline using the cluster approach to strengthen humanitarian response. 2006.

[41] Braun V, Clarke V (2006). Using thematic analysis in psychology. Qual Res Psychol.

[42] O’Brien BC, Harris IB, Beckman TJ, Reed DA, Cook DA (2014). Standards for reporting qualitative research: a synthesis of recommendations. Acad Med.

[43] The Technical and Operational Performance Support (TOPS) Technical and Operational Performance Support Program. Care groups: a reference guide for practitioners. Washington, DC: The Technical and Operational Performance Support Program. 2016.

[44] Phillips RM, Vujcic J, Boscoe A, Handzel T, Aninyasi M, Cookson ST (2015). Soap is not enough: handwashing practices and knowledge in refugee camps, Maban County. S Sudan Confl Health.

[45] Lam A (2000). Tacit knowledge, organizational learning and societal institutions: an integrated framework. Organ Stud.

[46] Caballero-Anthony M, Cook ADB, Chen C (2021). Knowledge management and humanitarian organisations in the Asia-Pacific: practices, challenges, and future pathways. Int J Disaster Risk Reduct.

[47] Shusterman J (2019). Method in the madness? Some new ways to learn from staff experiences in humanitarian crises: the historical case of UNICEF. Knowl Manag Dev J.

[48] Kruke BI, Olsen OE (2012). Knowledge creation and reliable decision-making in complex emergencies. Disasters.

[49] Sundberg M (2019). Donors dealing with ‘aid effectiveness’ inconsistencies: national staff in foreign aid agencies in Tanzania. J East Afr Stud.

[50] Daud Y (2021). Localisation of aid - the future of non-profit leadership in Africa: a review of the literature. PAC Univ J Arts and Soc Sci.

[51] Ward P (2020). Capitalising on ‘local knowledge’: the labour practices behind successful aid projects – the case of Jordan. Curr Sociol.

[52] Barbelet V (2019). Rethinking capacity and complementarity for a more local humanitarian action.

[53] Parrish C, Kattakuzhy A (2018). Money Talks: A synthesis report assessing humanitarian funding flows to local actors in Bangladesh and Uganda.

[54] White S, Heath T, Ibrahim WK, Ihsan D, Blanchet K, Curtis V, et al. How does hygiene behaviour change over the course of displacement? A qualitative case study in Iraq and Kurdistan. PloS One. In Press. 10.1371/journal.pone.0264434 PMC8893612 35239702

[55] White S, Mutula AC, Buroko MM, Heath T, Mazimwe FK, Blanchet K, et al. How does handwashing behaviour change in response to a cholera outbreak? A qualitative case study in the Democratic Republic of the Congo. In Press 10.1371/journal.pone.0266849 PMC9004767 35413080

[56] Gair S (2011). Feeling their stories: contemplating empathy, insider/outsider positionings, and enriching qualitative research. Qual Health Res.

[57] Tinker C, Armstrong N. From the outside looking in: how an awareness of difference can benefit the qualitative research process. 2008.

[58] Orford A (1999). Muscular humanitarianism: reading the narratives of the new interventionism. Eur J Int Law.

[59] Ticktin M (2014). Transnational humanitarianism. Annu Rev Anthropol.

[60] Fassin D (2011). Humanitarian reason: a moral history of the present.

