# Supplementary material for: How are hygiene programmes designed in crises? Qualitative interviews with humanitarians in the Democratic Republic of the Congo and Iraq
Source: Confl Health. 2022 Sep 2;16:45. doi: 10.1186/s13031-022-00476-8 (PMC9438112; doi:10.1186/s13031-022-00476-8)
Supplement: Supplementary file 1 — Additional file 1: Alignment with the Standards for Reporting Qualitative Research. [file 13031_2022_476_MOESM1_ESM.docx]

# Supplementary Material 1:

# Alignment with the Standards for Reporting Qualitative Research (1)

| **Title and Abstract** | |
| --- | --- |
| Title | The title includes a description of the topic of the research and mentions that it is a qualitative study using interviews. |
| Abstract | The abstract is structured to clearly present the study background, methods, results and conclusions. |
| **Introduction** | |
| Problem Formulation | The introduction provides a summary of the value of the research and the current gaps in evidence and practice. It also introduces relevant other research and theories that are applied to this study. |
| Purpose or Research Question | A specific study objective is stated at the end of the introduction. |
| **Methods** | |
| Qualitative approach and research paradigm | We specify that this work is grounded in a constructivist research paradigm and uses a comparative case study approach. |
| Researcher characteristics and reflexivity | In our methods we describe the characteristics of the data collection team. We reflect on the influence our positionality may have had on the findings and interpretation in the limitations section of the manuscript. |
| Context | We provide a description of the study sites and a rationale for their selection. |
| Sampling strategy | We provide a description of how participants were sampled and the basis for reaching a point of saturation. |
| Ethical issues pertaining to human subjects | We provide information on how consent was sought, what this covered and details about the ethical boards who reviewed this work. |
| Data collection methods | We provide details about when data was collected and how. |
| Data collection instruments and technologies | We describe how our interview guide was developed and how this was informed by theories and frameworks. |
| Units of study | In the results section we provide a summary of the participant characteristics. |
| Data processing | We mention that interviews were audio recorded, translated and transcribed and the development of a coding frame based on the conceptual frameworks used. |
| Data analysis | We describe the analysis approach used and how this involved multiple phases to verify data and then apply the coding frame and conceptual frameworks. |
| Techniques to enhance trustworthiness | We describe the participatory workshops that were used as an initial validation of the findings. We also describe how authors contributed to the validation of findings. |
| **Results/findings** | |
| Synthesis and interpretation | We structure our results according to the conceptual framework used and then compare some of the decision-making data to specific frameworks related to this within the humanitarian sector. |
| Links to empirical data | We provide quotes and examples from participants throughout the results in order to support our findings. |
| **Discussion** | |
| Integration with prior work, implications, transferability, and contributions to the field | We describe our key findings and how these are consistent with prior research but also add new evidence. |
| Limitations | We outline a range of limitations associated with the study. |
| **Other** | |
| Conflicts of Interest | We reflected on our conflicts of interest but had none to declare |
| Funding | We describe our funding source |

1. O’Brien BC, Harris IB, Beckman TJ, Reed DA, Cook DA. Standards for Reporting Qualitative Research: A Synthesis of Recommendations. Academic Medicine. 2014;89(9).
